# Synthesis and Analysis of Some Bis-Heterocyclic Compounds Containing Sulphur

**DOI:** 10.3390/molecules14051904

**Published:** 2009-05-22

**Authors:** Yahia Nasser Mabkhoot

**Affiliations:** Department of Chemistry, King Saud University P.O Box 2455, Riyadh-11451, Saudi Arabia; E-mail: yahia@ksu.edu.sa

**Keywords:** DMF-DMA, bis-thieno[2,3-b]thiophene, bis-aminopyrazole, bis-pyrazolopyrimidine

## Abstract

A facile and convenient synthesis of a series of bisheterocycles **7a,b 10, 12** and **13a,b** containing a thieno[2,3-b]thiophene base unit via the versatile, hitherto unreported 3-[3,4-dimethyl-5-(3-nitrilopropanoyl)thieno[2,3-b]thiophen-2-yl]-3-oxopropanenitrile (**4**) is described.

## Introduction

Thiophene derivatives represent a class of important and well-studied heterocycles [1,2]. The interest in this kind of heterocycles has spread from the early dye chemistry to modern drug design, biodiagnostics, electronic and optoelectronic devices, conductivity-based sensors, and self-assembled superstructures [3,4,5,6,7,8]. Recently, some conjugated thieno-thiophenes, structurally related to several current applications have been reported [9,10,11,12,13,14,15].

Thienothiophene derivatives have been developed for different purposes in the pharmaceutical field and have been tested as potential antitumor, antiviral, antibiotic, and antiglaucoma drugs, or as inhibitors of platelet aggregation [16,17,18,19,20]. Recently Mashraqui [10] described the first application of thieno[2,3-b]thiophene in the design of a novel NLO system by incorporating this nucleus within an unsymmetrically functionalized cyclophane. On the other hand, pyrazolopyrimidine compounds have been found to be useful as pharmaceutically interesting compounds [21,22]. 

However, little is known in the literature about thienothiene derivatives with different features and applications, and there is no report of a generally useful synthesis of thienothiene derivatives, compounds which are of considerable interest as potential biological active compounds or pharmaceuticals. In light of these findings we report here the synthesis of some novel bis-heterocycles containing thieno[2,3-b]thiophene as a base unit. 

## Results and Discussion

**Scheme 1:**
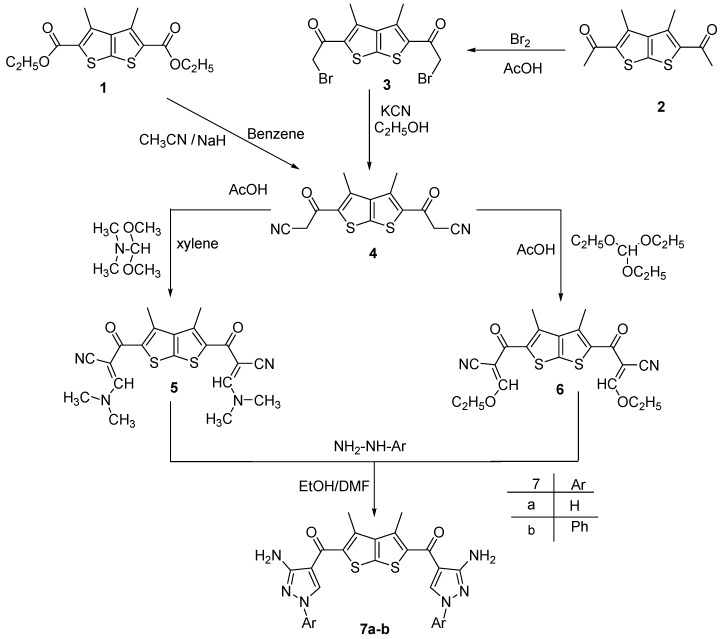
Synthesis of bis-aminopyrazoles derivatives **7a-b**.

**Scheme 2:**
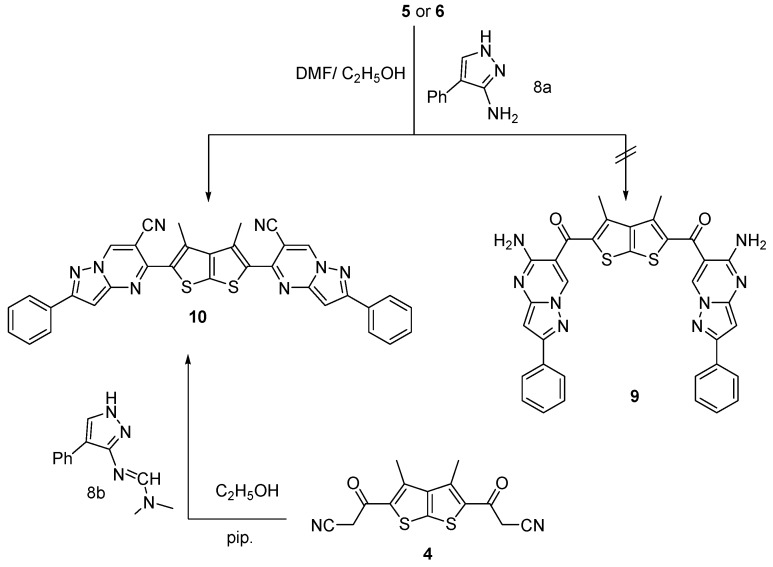
Synthesis of bis-pyrazolo[1,5-*a*]pyrimidine derivative **10**.

**Scheme 3:**
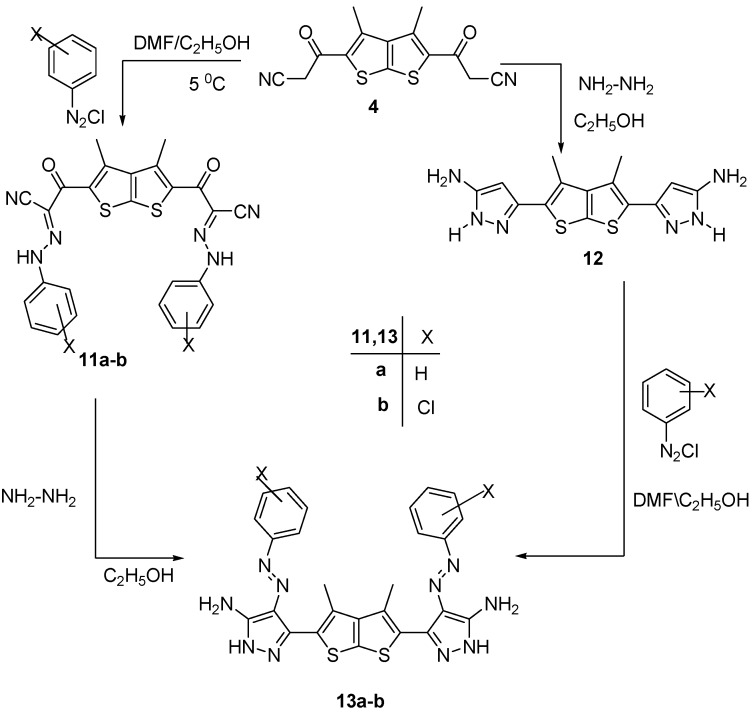
Synthesis of bis-amino pyrazole derivatives **13a,b**.

The synthetic procedures adopted to obtain the target compounds are depicted in Scheme 1, Scheme 2 and Scheme 3. Treatment of 3,4-dimethylthieno[2,3-b]thiophene-2,5-dicarboxylate (**1**) with acetonitrile in the presence of sodium hydride in refluxing benzene afforded the novel 3-[3,4-dimethyl-5-(3-nitrilopropanoyl)thieno[2,3-*b*]thiophen-2-yl]-3-oxopropanenitrile (**4**) (Scheme 1). 

The latter could alternatively be obtained by reaction of bis-2-bromoacetylthieno[2,3-b]thiophene derivative **3** with ethanolic potassium cyanide solution. Compound **3** in turn can be obtained from 1-(5-acetyl-3,4-dimethythieno[2,3-b]thiophene-2-yl)ethanone (**2**) upon treatment of Br_2_ in AcOH. The structure of compound **4** was established on the basis of its elemental analyses and spectral data. Its ^1^H- NMR spectrum displayed a singlet signal at δ 4.03 ppm, characteristic of active methylene protons, whereas its IR spectrum revealed two absorption bands at 2,214 and 1,709 cm^-1^ due to nitrile and carbonyl functions, respectively.

Treatment of compound **4** with dimethylformamide-dimethylacetal (DMF-DMA) in refluxing xylene afforded 2-({5-[-2-cyano-3-(dimethylamino)-2-propenoyl]-3,4-dimethylthieno[2,3-*b*]thiophen-2-yl}carbonyl)-3-(dimethylamino)-2-propenenitrile (**5**) (Scheme 1). The ^1^H-NMR spectrum of compound **5** displayed a singlet signal at δ 2.23 due to methyl protons, a singlet signal at δ 2.47 due to *N,N*-dimethyl protons and a singlet signal at δ 7.51 due to ethylenic proton. The mass spectrum revealed a molecular ion peak at m/z 412, corresponding to C_20_H_20_N_4_O_2_S_2_. In a similar manner, when **4** was treated with excess triethylorthoformate, the corresponding bis-ethoxypropenenitrile **6** was obtained in high yield. When compounds **5** and **6** were treated with hydrazine hydrate and with phenyl hydrazine in refluxing ethanol/DMF, the novel bis(aminopyrazoles) **7a,b**, were obtained, respectively (Scheme 1). The structures of the latter products were deduced from their elemental analyses and spectral data. The ^1^H-NMR spectrum of compound **7a**, for example, revealed a singlet signal at δ 7.52, characteristic of a pyrazole CH proton. 

Prompted by the aforementioned results, we have also investigated the reactivity of **5** and **6** towards 5-amino-3-phenyl-1H-pyrazole (**8a**). Thus, reaction of **5** and **6** with this compound in refluxing ethanol/DMF, in the presence of piperidine gave 6-[5-(7-cyanopyrazolo[1,5-*a*]pyrimidin-6-yl)-3,4-dimethylthieno[2,3-*b*]thiophen-2-yl]-2-phenylpyrazolo[1,5-*a*]pyrimidine-7-carbonitrile (**10**) (Scheme 2). The structure of the obtained product was assigned as **10** and not the other expected derivative **9** based on spectral data. The IR spectrum of the reaction product revealed, in each case, no bands due to amino or carbonyl functions. Moreover, the ^1^H NMR spectrum of compound **10**, revealed two singlet signals at δ 7.79 and 8.9 due to pyrazole and pyrimidine CH protons, respectively. The formation of product **10** is assumed to take place *via* the addition of the exocyclic amino group in 5-amino-3-phenyl-1H-pyrazole (**8a**) to the activated double bond in compounds **5 or 6** which then undergo intramolecular cyclization and subsequent aromatization *via* the loss of dimethylamine and water molecules under the reaction conditions to give **10** as depicted in Scheme 2.

Further evidence for the proposed structure **10** was obtained by treatment of 5-(*N,N* dimethylaminomethylene)imino-3-phenyl-1H-pyrazole (**8b**) [23] with **4** in ethanol/DMF in the presence of a catalytic amount of piperidine to afford a product indentical in all respects with that obtained from the reaction of **5** or **6** with 5-amino-3-phenyl-1H-pyrazole (Scheme 2).

Reactions of **4** with aromatic diazonium salts were also investigated. Thus, treatment of bis-3-oxopropanenitrile **4** with diazotized aromatic amines (aniline and *p*-chloroaniline) in cold ethanol/DMF, in the presence of sodium acetate trihydrate afforded the corresponding hydrazone derivatives **11a,b** (Scheme 3). 

The ^1^H-NMR spectrum of **11a**, for example, displayed besides an aromatic multiplet at δ 6.40-7.1 ppm, singlet signals at δ 2.22 and 9.20 ppm corresponding to methyl and hydrazone NH protons, respectively. Also, the IR spectra showed, in each case, absorption bands at 3200, 2220 and 1716 cm^-1^ due to NH, CN and CO groups, respectively. The latter hydrazones **11a,b** underwent intramolecular cyclization upon treatment with hydrazine hydrate to give products identified as the 3-aminopyrazole derivatives **13a,b**. The IR spectrum of **11a,b** showed, in each case, the absence of nitrile and carbonyl bands and revealed the appearance of three bands in the 3340-3100 cm^-1^ region due to NH_2_ and NH groups as depicted in Scheme 3. 

The ^13^C-NMR spectrum of **13a**, for example, revealed twelve carbon types. Its ^1^H-NMR spectrum displayed singlets at δ 4.21 and 12.7 ppm attributable to the NH_2_ and NH protons, respectively. Compounds **13a,b** were alternatively obtained by reaction of treatment of bis(aminopyrazole) derivative **12** with diazotized aromatic amines (aniline and *p*-chloroaniline) in cold ethanol/DMF, in the presence of sodium acetate trihydrate. Compound **12** was prepared by the reaction of **4** with hydrazine hydrate in refluxing ethanol (Scheme 3)**.**

## Experimental

### General

All melting points were measured on a Gallenkamp melting point apparatus. IR spectra were measured as KBr pellets on a Pye-Unicam SP 3-300 spectrophotometer. The NMR spectra were recorded on a Varian Mercury VX-300 NMR spectrometer. ^1^H-NMR (300 MHz) and ^13^C-NMR (75.46 MHz) were run in dimethylsulphoxide (DMSO-d_6_). Chemical shifts were related to that of the solvent. Mass spectra were recorded on a Shimadzu GCMS-QP 1000 EX mass spectrometer at 70 eV. Elemental analysis was carried out on an Elementar Vario EL analyzer. Thieno[2,3-b]thiophene derivatives **1**,**2** were prepared following literature procedures [24,25]. 

### 2-Bromo-1-[5-(2-bromoacetyl)-3,4-dimethylthieno[2,3-b]thiophen-2-yl]-ethanone (***3**)*

A solution of **2** (25.2 g, 100 mmol) in glacial acetic acid (100 mL) was heated to 80–90 °C with vigorous stirring. To this hot solution, bromine (16 g, 100 mmol) in glacial acetic acid (20 mL) was added dropwise over a period of 30 min. After complete addition of bromine, the reaction mixture was stirred vigorously at room temperature for further 1 h till the evolution of hydrogen bromide gas ceased, then it was poured onto crushed ice. The solid that formed was collected, washed with water, dried well and recrystallized from ethanol to give colorless crystals of **3**; yield 87%, mp. 180 –182 °C; IR (KBr) ν_max_ 1690 (C=O) cm^-1^; ^1^H-NMR: δ 2.23 (s, 6H, CH_3_), 4.82 (s, 4H, CH_2_); ^13^C-NMR: δ 9.3 (CH_3_, aliphatic), 28.50 (CH_2_), 132.3, 136.1, 140.2, 148.4 (ArC’s), 186.2 (C=O). MS m/z (%): 407 (M^+^, 100) , 286 (15), 249 (28), 116 (74); Anal. Calcd. for C_12_H_10_Br_2_O_2_S_2_ (407.85): C, 35.14; H, 2.46; Br, 38.96; S,15.64. Found: C, 35.03; H, 2.52; Br, 38.85; S, 15.54.

### 3-[3,4-Dimethyl-5-(3-nitrilopropanoyl)thieno[2,3-b]thiophen-2-yl]-3-oxopropanenitrile *(**4**)*

Route A: To a mixture of 3,4-dimethylthieno[2,3-b]thiophene-2,5-dicarboxylate (**1**, 31.24 g, 100 mmol) in dry benzene (200 mL) and dimethylformamide (10 mL) was added sodium hydride (2.5 g, 60%). The reaction mixture was refluxed for 4h, then allowed to cool. The solid that precipitated was collected, washed with ether and dried. The product was dissolved in water and the resulting solution was treated with concentrated hydrochloric acid until it becomes neutral. The precipitated product was collected, washed with water, dried and finally recrystallized from DMF/EtOH as colorless crystals; yield 75%, mp. 254 –256 °C; IR (KBr) ν_max_ 1709 (C=O), 2214 (CN) cm^-1^; ^1^H-NMR: δ 2.23 (s, 6H, CH_3_), 4.03 (s, 4H, CH_2_); ^13^C-NMR: δ 9.3 (2CH_3_, aliphatic), 28.50 (2CH_2_), 116.3 (2CN), 131.7, 134.5, 143.4, 160.5 (ArC’s), 193.2 (2 C=O); MS m/z (%): 303 (M^+^ +1, 100), 302 (M^+^, 85), 286 (17), 249 (19), 160 (34); Anal. for C_14_H_10_N_2_O_2_S_2_ (302.37) calcd.: C, 55.61; H, 3.33; N, 9.26; S, 21.21. Found: C, 55.47; H, 3.22; N, 9.30 ; S, 21.20.

Route B: Compound **3** (41.01 g, 100 mmol) in ethanol (15 ml) was treated with a solution of KCN (6.7 g, 100mmole) under reflux for 1h. The resulting mixture was poured onto cold water. The solid precipitate was filtered, dried and recrystallized from DMF/EtOH as colorless crystals, yield 85%. This compound was identical in all respects (mp., mixed mp and spectral data) with compound **3** obtained by route A above.

### 2-({5-[2-Cyano-3-(dimethylamino)-2-propenoyl]-3,4-dimethylthieno[2,3-b]thiophen-2-yl}carbonyl)-3-(dimethylamino)-2-propenenitrile *(**5**)*

A mixture of bis-3-oxopropanenitrile **4** (20 mmol) and dimethylformamide–dimethylacetal (DMF–DMA, 20 mmol) in dry xylene (20 mL) was refluxed for 8 h, then left to cool to room temperature. The reddish-brown precipitated product was filtered off, washed with light petroleum (40–60 °C), and dried. Recrystallization from DMF/EtOH afforded **5**. Reddish-brown crystals; Yield 70%, mp. 290 - 292 °C; IR (KBr) ν_max_, 1713 (C=O), 2221(CN), 1554 (C=C) cm^-1^; ^1^H-NMR: δ 2.23 (s, 6H, CH_3_), 2.47 (s, 12H, CH_3_), 7.51 (s, 2H, CH); ^13^C-NMR: δ 9.2 (2CH_3_, aliphatic), 42.7 (4CH_3_), 85.2 (2C=), 115.9 (2CN), 131.2, 136.1, 138.1, 147.4 (ArC’s), 157.9 (2CH=) 193.2 (2C=O); MS m/z (%): 413 (M^+^ +1, 76), 412 (M^+^,100), 286 (22), 249 (13); Anal. for C_20_H_20_N_4_O_2_S_2_ (412.53) calcd.; C, 58.23; H, 4.89; N, 13.58; S, 15.55. Found: C, 58.12; H, 4.64; N, 13.53; S, 15.49.

### 2-({5-[2-Cyano-3-ethoxy-2-propenoyl]-3,4-dimethylthieno[2,3-b] thiophen-2-yl}carbonyl)-3-ethoxy-2-propenenitrile *(**6**)*

A mixture of bis-3-oxopropanenitrile **4** (10 mmol), triethylorthoformate (3 mL) and glacial acetic acid (3 mL) was refluxed for 3 h. The solid precipitate that formed was filtered off, washed with ethanol, dried and finally recystallized from DMF/EtOH afford **5**. Yellow crystals; yield 83%, mp. 282–283 °C; IR (KBr) ν_max_ 1720 (C=O), 2205 (CN), 1552 (C=C) cm^-1^; ^1^H-NMR: δ 1.22 (tr, 6H, CH_3_ ) 2.23 (s, 6H, CH_3_), 4.02 (q, 4H, CH_2_),7.93 (s, 2H, CH); ^13^C-NMR: δ 9.2 (2CH_3_, aliphatic), 15.3 (2CH_3_), 64.0 (2CH_2_), 80.1 (2C=), 115.9 (2CN), 132.2, 136.7, 138.9, 147.5, (ArC’s), 155.4 (2CH=), 189.7 (2C=O); MS m/z (%): 415.5 (M^+^ +1, 100), 414 (M^+^, 76), 286 (30), 249 (20); Anal. for C_20_H_18_N_2_O_4_S_2_ (414.5) calcd.; C, 57.95 ; H, 4.38; N, 6.76 ; S, 15.47. Found: C, 57.95; H, 4.38; N, 6.76; S, 15.47.

### General procedure for the reaction of bis-thieno[2,3-b]thiophene derivatives ***5, 6*** with hydrazine derivatives

Treatment of compounds **5** or **6** (1 mmol) with hydrazine hydrate and with phenyl hydrazine (0.1 mL) in dry ethanol (15 mL) under reflux for 5 h afforded the corresponding derivatives **7a,b**, respectively. The solid products so formed was filtered off, washed with ethanol, dried and recrystallized from DMF/EtOH).

*(3-Amino-1H-pyrazol-4-yl){5-[(3-amino-1H-pyrazol-4-yl)carbonyl]-3,4-dimethylthieno[2,3-b]thio-phen-2-yl}methanone* (**7a**): Colorless crystals; Yield (82%); mp >300 ^0^C; IR (KBr) ν_max_ 3320, 3275, 3100 (NH_2_+NH), 1735 (C=O), 1543 (CH=C) cm^-1^; ^1^H-NMR: δ 2.22 (s, 6H, CH_3_), 4.92 (s, 4H, NH_2_), 7.52 (s, 2H, pyrazole), 9.42 (s, 2H, NH); ^13^C-NMR: δ 9.3 (2CH_3_, aliphatic), 94.1, 127.6, 131.2, 136.1, 141.0, 147.5, 161.0 (ArC’s),183.7 (C=O); MS m/z (%): 387 (M^+^ +1,100) 386 (M^+^,20) 285 (30), 251 (20); Anal. for C_16_H_14_N_6_O_2_S_2_ (386.45) calcd.; C, 49.73; H, 3.65; N, 21.75; S, 16.59. Found: C, 49.57; H, 3.41; N, 21.55; S, 16.64.

*(3-Amino-1-phenyl-1H-pyrazol-4-yl){5-[(3-amino-1-phenyl-1H-pyrazol-4-yl)carbonyl]-3,4-dimethyl-thieno[2,3-b]thiophen-2-yl}methanone* (**7b**): Reddish-brown crystals; Yield (70%); mp >300 ^0^C; IR (KBr) ν_max_ 3320, 3275 (NH_2_), 1720 (C=O), 1538 (CH=C) cm^-1^; ^1^H-NMR: δ 2.22 (s, 6H, CH_3_), 4.92 (s, 4H, NH_2_), 7.52 (s, 2H, CH pyrazole), 7.3 (m, 10H ArH’s); ^13^C-NMR: δ 9.3 (2CH_3_, aliphatic), 94.1, 120.2, 126.3, 127.6, 129.4, 131.2, 136.1, 139.7, 141.0, 147.5, 161.0 (ArC’s), 185.0 (2C=O); MS m/z (%): 539 (M^+^ +1, 55), 538 (M^+^, 100), 284 (30), 117 (20); Anal. for C_28_H_22_N_6_O_2_S_2_ (538.64) calcd.; C, 62.43; H, 4.12; N, 15.60; S, 11.91. Found: C, 62.39; H, 4.04; N, 15.75; S, 11.78.

### 6-[5-(7-Cyanopyrazolo[1,5-a]pyrimidin-6-yl)-3,4-dimethylthieno[2,3-b]thiophen-2-yl]-2-phenyl-pyrazolo[1,5-a]pyrimidine-7-carbonitrile *(**10**)*

Method A: To a mixture of the bis-enaminone **5** or bis-ethoxypropenenitrile **6** (10 mmol) and 5-amino-3-phenyl-1H-pyrazole (**8a)** (10 mmol) in DMF/EtOH (25 mL) was added few drops of piperidine and the reaction mixture was refluxed for 3 h, then left to cool. The formed solid product was filtered off and recrystallized from EtOH/DMF to afford the pyrazolo-[1,5-a]pyrimidine derivatives **10** in 75 % yield; ^1^H-NMR: δ 2.22 (s, 6H, CH_3_), 7.36-7.61 (m, 10H, Ph), 7.79 (s, 2H, pyrazole), 8.9 (s, 2H, pyrimidine); ^13^C-NMR: δ 9.3 (2CH_3_, aliphatic), 118.2 (2CN), 92.7, 106.5, 127.6, 125.5, 127.2, 129.1, 130.2, 130.8, 134.5, 138.6, 148.3, 150.3, 154.2 (ArC’s); MS m/z (%): 604 (M^+^,100), 284 (29), 253 (25), 115 (61); Anal. for C_34_H_20_N_8_S_2_ (604.71) calcd.; C, 67.53; H, 3.33; N, 18.53; S, 10.61. Found: C, 67.68; H, 3.42; N, 18.81; S, 10.77.

Method B: A solution of 3-[3,4-dimethyl-5-(3-nitrilopropanoyl)thieno[2,3-*b*]thiophen-2-yl]-3-oxopropanenitrile (**4**, 10 mmol) and an equivalent molar ratio of 5-N-(N,N-dimethylaminomethylene)-amino-3-methyl-1H-pyrazole (**8b**) in ethanol (20 mL), in the presence of piperidine (0.3 mL), was heated under reflux for 6 h. The solvent was removed by distillation under reduced pressure and the remainder was left to cool. The precipitated solid product was collected by filtration. Recrystallization from EtOH/DMF afforded a product identical in all respects (mp, mixed mp, TLC, IR, and mass spectra with **10.**

### 2-Arylhydrazono-3-dimethylthieno[2,3-b]thiophen-3-oxopropanenitriles ***11a,b***

To a stirred cold solution of bis-3-oxopropanenitrile **4** (6.04 g, 20 mmol) in EtOH/DMF (30 mL) and sodium acetate trihydrate (2 g), was added the appropriate arene diazonium chloride (20 mmol) portionwise over a period of 30 min at 0-5°C. After complete addition, the reaction mixture was stirred for further 3h at 0-5°C. The solid that precipitated was collected, washed with water and dried. Recrystallization from EtOH/DMF afforded the corresponding hydrazones **11a,b**.

*3-{5-[5-Amino-4-(2-phenylhydrazino)-1H-pyrazol-3-yl]-3,4-dimethylthieno[2,3-b]thiophen-2-yl}-4-(2-phenylhydrazino)-1H-pyrazo-5-yl amine* (**11a**): Yellow crystals; Yield (80%); mp. 275^0^C; IR (KBr) ν_max_ 3200 (NH), 2977, 2220 (CN), 1716 (C=O), 1533 (CH=C) cm^-1^; ^1^H-NMR: δ 2.22 (s, 6H, CH_3_), 6.40 -7. 10 (m, 10H, ArH’s), 9.20 (s, 2H, NH); ^13^C-NMR: δ 9.3 (2CH_3_, aliphatic), 115.2 (2CN), 117.6, 128.2, 129.8, 132.2, 136.0, 138.2, 142.0, 148.4, (ArC’s), 187.2 (2C=O). MS m/z (%): 511 (M^+^ +1, 100) 510 (M^+^, 60) 286 (29), 250 (25); Anal. for C_26_H_18_N_6_O_2_S_2_ (510.59) calcd.; C, 61.16; H, 3.55; N, 16.46; S, 12.56. Found: C, 60.97; H, 3.64; N, 16.42; S, 12.42.

*2-[2-(4-Chlorophenyl)hydrazono]-3-(5-{2-[2-(4-chlorophenyl)hydrazono]-3-nitrilopropanoyl}-3,4-dimethylthieno[2,3-b]thiophen-2-yl)-3-oxopropanenitrile* (**11b**): Yellow crystals; Yield (85%); mp. 283^0^C; IR (KBr) ν_max_ 3225 (NH), 2220 (CN), 1707 (C=O), 1521 (CH=C) cm^-1^; ^1^H-NMR: δ 2.22 (s, 6H, CH_3_), 6.24 -7. 17 (m, 8H, ArH’s), 8.21 (s, 2H, NH); ^13^C-NMR: δ 9.2 (2CH_3_, aliphatic), 114.9 (2CN), 117.7, 124.3, 129.7, 131.2, 136.1, 138.1, 141.2, 147.5 (ArC’s), 184.0 (2C=O); MS m/z (%): 580 (M^+^ +1, 100), 579 (M^+^, 20), 286 (35), 117 (25); Anal. for C_26_H_16_Cl_2_N_6_O_2_S_2_ (579.48) calcd.; C, 53.89; H, 2.78; Cl, 12.24; N ,14.50; S, 11.07. Found: C, 53.68; H, 2.69; Cl, 12.52; N, 14.63; S, 10.88.

### 3-[5-(5-Amino-1H-pyrazol-3-yl)-3,4-dimethylthieno[2,3-b]thiophen-2-yl]-1H-pyrazol-5-yl amine *(**12**)*

A mixture of the bis-3-oxopropanenitrile **4** (6.04 g, 20 mmol) and hydrazine hydrate (1 mL, 80%) in absolute ethanol (20 ml) was refluxed for 2-4 h. The reaction mixture was allowed to cool and then diluted with water. The precipitated solid was collected, washed with water and dried. Recrystallization from EtOH/DMF afforded the pyrazole derivative **12.** Colorless crystals; Yield (73%); mp.285 ^0^C; IR (KBr) ν_max_ 3320, 3250, 3120 (NH_2_+NH), 1543 (CH=C) cm^-1^; ^1^H-NMR: δ 2.21 (s, 6H, CH_3_), 4.02 (br, 4H, NH_2_), 6.03 (s, 2H, pyrazole), 12.98 (s, 2H, NH); ^13^C-NMR: δ 9.3 (2CH_3_, aliphatic), 88.8, 125.7, 130.2, 133.4, 135.9.0, 138.1.5, 153.0 (ArC’s). MS m/z (%): 331 (M^+^ +1,90), 330 (M^+^, 76) 286 (100), 249 (45); Anal. for C_14_H_14_N_6_S_2_ (330.43) calcd.; C, 50.89; H, 4.27; N, 25.43 ; S, 19.41. Found: C, 50.76; H, 4.26; N, 25.43; S, 19.46.

### 5-Amino-4-arylazo-3-(thieno[2,3-b]thiophen-2-yl)pyrazoles ***13a,b***

Route A: To a solution of the appropriate hydrazone **11a,b** (5 mmol) in ethanol (20 mL) was added hydrazine hydrate (5 mmol). The reaction mixture was refluxed for l h, then cooled. The solid formed was collected, washed with ethanol, dried and finally recrystallized from EtOH/ DMF to afford the corresponding 4-arylazopyrazole derivatives **13a,b**, respec- tively.

Route B: To a stirred cold solution of bis-pyrazole derivative **12** (6.60 g, 20 mmol) in EtOH/DMF (30 mL) and sodium acetate trihydrate (2 g), was added the appropriate arene diazonium chloride (20 mmol) portionwise over a period of 30 min at 0-5°C. After complete addition, the reaction mixture was stirred for further 3 h at 0-5°C. The solid that precipitated was collected, washed with water and dried. Recrystallization from EtOH/DMF afforded the corresponding hydrazones **13a,b**.

*4-(2-Phenyl)diazenyl)-3-(2-(4-(2-phenyl)diazenyl)-5-amino-1H-pyrazol-3-yl)-3,4-dimethylthieno[2,3-b]thiophen-2-yl) )-1H-pyrazol-5-amine* (**13a**): Colorless crystals; Yield (45%); mp. >300 ^0^C; IR (KBr) ν_max_ 3306, 3244, 3100 (NH_2_+NH) cm^-1^; ^1^H-NMR: δ 2.22 (s, 6H, CH_3_), 4.21 (br, 4H, NH_2_), 6.54 -7.17 (m, 10H, ArH’s), 12.7 (s, 2H, NH); ^13^C-NMR: δ 9.3 (2CH_3_, aliphatic), 92.1, 117.2, 121.6, 125.7, 128.5, 128.9, 132.1, 133.8, 138.4, 143.4, 154.0 (ArC’s); MS m/z (%): 539 (M^+^ +1,65), 538 (M^+^,100), 283 (29), 251 (25); Anal. for C_26_H_22_N_10_S_2_ (538.65) calcd.; C, 57.97; H, 4.12; N, 26.00; S, 11.91. Found: C, 57.70; H, 4.18; N, 26.15; S, 11.69.

*4-(2-(4-Chlorophenyl)diazenyl)-3-(2-(4-(2-(4-chlorophenyl)diazenyl)-5-amino-1H-pyrazol-3-yl)-3,4-dimethylthieno[2,3-b]thiophen-2-yl) )-1H-pyrazol-5-amine* (**13b**)**:** Colorless crystals; Yield (60%); mp. >300 ^0^C; IR (KBr) ν_max_ 3333, 3221, 3150 (NH_2_+NH) cm^-1^; ^1^H-NMR: δ 2.22 (s, 6H, CH_3_), 4.00 (br, 4H, NH_2_), 7. 20-7.32 (m, 8H, ArH’s), 13.7 (s, 2H, NH); ^13^C-NMR: δ 9.3 (2CH_3_, aliphatic), 92.1, 125.8, 126.6, 125.7, 128.5, 130.2, 130.3, 133.7, 134.3, 138.4, 154.0, (ArC’s); MS m/z (%): 608 (M^+^ +1, 100), 607 (M^+^,54), 286 (29), 115 (25); Anal. for C_26_H_20_Cl_2_N_10_S_2_ (607.54) calcd.; C, 51.40; H, 3.32; Cl, 11.67; N, 23.05; S, 10.56. Found: C, 51.78; H, 3.26; Cl, 11.84; N, 23.17; S, 10.55.

## Conclusions

Synthesis and identification of some bis-heterocycles **7a,b, 10, 12** and **13a,b** containing thieno[2,3-b]thiophene as a base unit via the versatile, hitherto unreported 3-[3,4-dimethyl-5-(3-nitrilopropanoyl)thieno[2,3-b]thiophen-2-yl]-3-oxopropanenitrile(**4**) was reported.

## References

[1] Eicher T., Hauptmann S., Speicher A. (2003). Five-Membered Heterocycle. The Chemistry of Heterocycles.

[2] Gronowitz S., Gronowitz S. (1991). The Chemistry of Heterocyclic Compounds: Thiophene and its Derivatives.

[3] King W.J., Nord F.F. (1949). Studies In The Thiophene Series. V. Wolff-Kishner Reductions. J. Org. Chem..

[4] Wu C., Decker E.R., Blok N., Bui H., You T.J., Wang J., Bourgoyne A.R., Knowles V., Berens K.L., Holland G.W., Brock T.A., Dixon R.A.F. (2004). Discovery, Modeling, and Human Pharmacokinetics of *N*-(2-Acetyl-4,6-dimethylphenyl)-3-(3,4-dimethylisoxazol-5-ylsulfamoyl)-thiophene-2-carboxamide (TBC3711), a Second Generation, ET_A_ Selective, and Orally Bioavailable Endothelin Antagonist. J. Med. Chem..

[5] Dubus S., Levesque I., Brunette M., Corbeil G., Boissinot M., Boivin G., Bergeron M.G., Boudreau D., Leclerc M. (2004). Fluorescent Polymeric Transducer for the Rapid, Simple, and Specific Detection of Nucleic Acids at the Zeptomole Level. J. Am. Chem. Soc..

[6] Rost C., Karg S., Riess W., Loi M.A., Murgia M., Muccini M. (2004). Ambipolar light-emitting organic field-effect transistor. Appl. Phys. Lett..

[7] Vriezema D.M., Hoogboom J., Velonia K., Takazawa K., Christianen P.C.M., Maan J.C., Rowan A.E., Nolte R.J. (2003). Vesicles and Polymerized Vesicles from Thiophene-Containing Rod-Coil Block Copolymers. Angew. Chem. Int. Ed..

[8] Pullen A.E., Bushel M.G., Swager T.M. (2004). Charge-Specific Interactions in Segmented Conducting Polymers: An Approach to Selective Ionoresistive Responses. Angew. Chem. Int. Ed..

[9] Heeney M., Bailey C., Genevicius K., Shkunov M., Sparrowe D., Tierney S., Mculloch I. (2005). Stable Polythiophene Semiconductors Incorporating Thieno[2,3-*b*]thiophene. J. Am. Chem. Soc..

[10] Mashraqui S.H., Sangvikar Y.S., Meetsma A. (2006). Synthesis and structures of thieno[2,3- b]thiophene incorporated [3.3]dithiacyclophanes. Enhanced first hyperpolarizability in an unsymmetrically polarized cyclophane. Tetrahedron Lett..

[11] Mashraqui S.H., Sangvikar Y., Ashraf M., Kumar S., Daub E. (2005). Dipyridyl/pyridinium thieno[2,3-b]thiophenes as new atropisomeric systems. Synthesis, conformat-ional analysis and energy minimization. Tetrahedron.

[12] Leriche P.R.J., Turbiez M.M., Monroche V., Allain M., Sauvage F.X., Roncali J., Frere P., Skabara P.J. (2003). Linearly extended tetrathiafulvalene analogues with fused thiophene units as π-conjugated spacers. J. Mater. Chem..

[13] Lee B., Seshadri V., Palko H., Sotzing G.A. (2005). Ring-Sulfonated Poly(thienothiophene. J. Adv. Mater..

[14] Lim E., Jung B.J., Lee J., Shim H.K., Lee J.I., Yang Y.S., Do L.M. (2005). Thin-Film Morphologies and Solution-Processable Field-Effect Transistor Behavior of a Fluorene−Thieno[3,2-*b*]thiophene-Based Conjugated Copolymer. Macromolecules.

[15] Kim H.S., Kim Y.H., Kim T.H., Noh Y.Y., Pyo S., Yi M.H., Kim D.Y., Kwon S.K. (2007). Synthesis and Studies on 2-Hexylthieno[3,2-b]thiophene End-Capped Oligomers for OTFTs. Chem. Mater..

[16] Jarak I., Kralj M., Piantanida I., Suman L., Zinic M., Pavelic K., Karminski-Zamola G. (2006). Novel cyano- and amidino-substituted derivatives of thieno[2,3-b]- and thien- o[3,2-b]thiophene-2-carboxanilides and thieno[30,20:4,5]thieno- and thieno [20,30:4,5] thieno[2,3-c]quinolones: Synthesis, photochemical synthesis, DNA binding, and antitumor evaluation. Bioorg. Med. Chem..

[17] Peters D., Hornfeldt A.B., Gronowitz S. (1990). Synthesis of various 5-substituted uracils. J. Heterocycl. Chem..

[18] Kukolja S., Draheim S.E., Graves B.J., Hunden D.C., Pfeil J.L., Cooper R.D.G., Ott J.L., Couter F.T. (1985). Orally absorbable cephalosporin antibiotics. 2. Structure-activity studies of bicyclic glycine derivatives of 7-aminodeacetoxycephalosporanic acid. J. Med. Chem..

[19] Prugh J.D., Hartman G.D., Mallorga P.J., McKeever B.M., Michelson S.R., Murcko M.A., Schwam H., Smith R.L., Sondey J.M., Springer J.P., Surgrue M.F. (1991). New isomeric classes of topically active ocular hypotensive carbonic anhydrase inhibitors: 5-substituted thieno[2,3-b]thiophene-2-sulfonamides and 5-substituted thieno[3,2-b]thiophene-2-sulfonamides. J. Med. Chem..

[20] Egbertson M.S., Cook J.J., Bednar B., Prugh J.D., Bednar R.A., Gaul S.L., Gould R.J., Hartman G.D., Homnick C.F., Holahan M.A., Libby L.A., Lynch J.J., Lynch R.J., Sitko G.R., Stranieri M.T., Vassallo L.M. (1999). Non-Peptide GPIIb/IIIa Inhibitors. 20. Centrally Constrained Thienothiophene α-Sulfonamides Are Potent, Long Acting in Vivo Inhibitors of Platelet Aggregation. J. Med. Chem..

[21] Schuett W., Raoort H. (1962). Saxitoxin, the Paralytic Shellfish Poison. Degradation to a Pyrrolopyrimidine. J. Am. Chem. Soc..

[22] Alexander S.K., Smith L. (2006). Novel one pot synthesis of polysubstituted pyrazolo[1,5-a] and imidazo[1,2-a]pyrimidines. Tetrahedron Lett..

[23] Shaaban M.R., Saleh T.S., Mayhoub A., Farag A.M. (2008). Synthesis and analgesic/ anti-inflammatory evaluation of fused heterocyclic ring systems incorporating phenylsulfonyl moiety. Bioorg. Med. Chem..

[24] Comel A., Kirsch G. (2001). Efficient one pot preparation of variously substituted thieno [2,3-b] thiophene. J. Heterocycl. Chem..

[25] El-Saghier M.A. (1993). Simple One-Pot Synthesis of Thieno[2,3-*b*]thiophene Derivatives under Solid–Liquid PTC Conditions. Useful Starting Material for the Synthesis of Biological Active Compounds. Bull. Chem. Soc. Jpn..

